# A Vanillin Derivative Causes Mitochondrial Dysfunction and Triggers Oxidative Stress in *Cryptococcus neoformans*

**DOI:** 10.1371/journal.pone.0089122

**Published:** 2014-02-20

**Authors:** Jin Hyo Kim, Han-Ok Lee, Yong-Joon Cho, Jeongmi Kim, Jongsik Chun, Jaehyuk Choi, Younghoon Lee, Won Hee Jung

**Affiliations:** 1 Chemical Safety Division, National Academy of Agriculture Science, Rural Development of Administration, Suwon, Republic of Korea; 2 Department of Systems Biotechnology, Chung-Ang University, Anseong, Republic of Korea; 3 ChunLab, Inc., Seoul National University, Seoul, Republic of Korea; 4 School of Biological Sciences, Seoul National University, Seoul, Republic of Korea; 5 Research Division for Biotechnology, Korea Atomic Energy Research Institute, Jeongup, Republic of Korea; 6 Department of Chemistry, KAIST, Daejeon, Republic of Korea; Yonsei University, Korea, Republic Of

## Abstract

Vanillin is a well-known food and cosmetic additive and has antioxidant and antimutagenic properties. It has also been suggested to have antifungal activity against major human pathogenic fungi, although it is not very effective. In this study, the antifungal activities of vanillin and 33 vanillin derivatives against the human fungal pathogen *Cryptococcus neoformans*, the main pathogen of cryptococcal meningitis in immunocompromised patients, were investigated. We found a structural correlation between the vanillin derivatives and antifungal activity, showing that the hydroxyl or alkoxy group is more advantageous than the halogenated or nitrated group in benzaldehyde. Among the vanillin derivatives with a hydroxyl or alkoxy group, *o*-vanillin and *o*-ethyl vanillin showed the highest antifungal activity against *C. neoformans*. *o*-Vanillin was further studied to understand the mechanism of antifungal action. We compared the transcriptome of *C. neoformans* cells untreated or treated with *o*-vanillin by using RNA sequencing and found that the compound caused mitochondrial dysfunction and triggered oxidative stress. These antifungal mechanisms of *o*-vanillin were experimentally confirmed by the significantly reduced growth of the mutants lacking the genes involved in mitochondrial functions and oxidative stress response.

## Introduction

The incidence rates of infectious diseases caused by pathogenic fungi have been increasing rapidly. Widespread fungal diseases not only affect plants but also threaten animal and human health, with morbidity and mortality critically influenced by superficial and invasive fungal infections [1]. Particular attention has been paid to invasive fungal infections, which have a high mortality rate of more than 50%. Each year, at least 1.5 million people die from invasive fungal infections worldwide [2]. The most frequently isolated human pathogenic fungi are *Cryptococcus*, *Candida*, and *Aspergillus* species, which normally cause diseases in patients with immunodeficiency due to human immunodeficiency virus (HIV) infections or immunosuppressive medications for organ transplantation. Among these commonly isolated fungal pathogens, *Cryptococcus neoformans* causes cryptococcal meningitis and kills at least 650,000 patients annually throughout the world. Cryptococcal infections have a reported mortality rate of 20–70% [3].

Currently available antifungal agents mostly target DNA and RNA synthesis, the biosynthesis of the cell wall component 1,3-β-d-glucan, the ergosterol biosynthetic pathway, or ergosterol itself. Triazole, which inhibits the ergosterol biosynthetic pathway, is the most widely used fungistatic agent because of its effectiveness and safety. Fluconazole has been used extensively to treat superficial and invasive fungal infections. However, the number of resistant strains to currently available antifungal agents has increased dramatically during the last decade [4], [5]. The mechanism of resistant strains is through increased drug efflux or alteration of the drug target or target pathway, which reduces drug efficacy [6]. Therefore, there is increasing demand for a novel compound to treat fungal infections.

Plant extracts have been used as an effective source of antifungal agents. Vanillin (4-hydroxy-3-methylbenzaldehyde) is a good example, which is a primary component of the vanilla bean extract. Vanillin has long been used as a flavoring compound and is generally recognized as safe. Each year, more than 12,000 tons of vanillin is consumed, but the compound is mainly synthetically produced because the naturally derived product is expensive [7]. Because of its safety and long-established use as a food additive, a number of studies have investigated the potential of vanillin as an antifungal agent. However, no report has shown promising efficacy of vanillin against fungi. Lopez-Malo et al. [8] and Cerrutti and Alzamora [9] suggested a possible use of vanillin as an antifungal agent in food preservation. However, its inhibitory concentration was too high to be promising. A recent study by Faria et al. [10] also found no growth inhibitory activity of vanillin on nine reference human pathogenic fungal strains of *Candida* and *C. neoformans*.

This study aimed to search for a vanillin derivative that has strong antifungal efficacy against *C. neoformans* and to understand the mechanism of action of the compound. We used *C. neoformans* because of its clinical importance, well-annotated genome sequence, and robust genetic tools. A series of vanillin derivatives, including hydroxy and alkoxy benzaldehydes, halogenated benzaldehydes, and nitrated benzaldehydes, were tested for their antifungal activity against *C. neoformans*. We found a structural correlation between the vanillin derivatives and antifungal activity against *C. neoformans*; that is, the hydroxyl or alkoxy group appeared to be more advantageous than the halogenated or nitrated group in benzaldehyde. Among the vanillin derivatives with a hydroxyl or alkoxy group, *o*-vanillin and *o*-ethyl vanillin showed the highest antifungal activities against *C. neoformans*. We chose *o*-vanillin to study the mechanism of action. To date, numerous studies have successfully used functional genomics approaches, in particular transcriptome analysis, to identify the target pathway of currently available antifungal drugs and novel antifungal candidate drugs [11]–[14]. We therefore used transcriptome analysis to understand the mechanism of action of *o*-vanillin. Global transcript profiles of *C. neoformans* cells treated with *o*-vanillin were obtained using RNA sequencing (RNA-Seq) and were compared with the transcript profiles of the same strain not treated with the compound. The results of our transcriptome analysis suggested that *o*-vanillin likely acts by significantly reducing mitochondrial function, which would in turn disrupt cellular redox-homeostasis in *C. neoformans*. Our hypothesis was experimentally confirmed by the observation of hypersensitivity of the *C. neoformans* mutants lacking the genes involved in the oxidative stress response upon treatment with *o*-vanillin.

## Materials and Methods

### Strains

The wild-type strain used in this study was *Cryptococcus neoformans* var. *grubii* H99. The *cfo1* mutant was constructed as described elsewhere [15]. The *sod1* mutant, *sod2* mutant, and *ccp1* mutant were constructed using biolistic transformation of the gene-specific knock-out cassettes, which were generated using overlapping polymerase chain reaction (PCR) with primers listed in Table S1 [16]. The positive mutants were selected by PCR, and at least two independent mutants were used throughout the study. Fungal cells were routinely grown in YPD medium (1% yeast extract, 2% bacto-peptone, and 2% glucose) at 30°C. Vanillin and the vanillin derivatives used in this study were purchased from Tokyo Chemical Industry (Tokyo, Japan).

### Antifungal Drug Sensitivity

To estimate sensitivity to the antifungal drug, the fungal cells were grown in 3 mL of YPD medium at 30°C overnight with shaking, and minimal inhibitory concentrations (MIC) were determined as described elsewhere [17]. To investigate the susceptibility of the wild-type and mutant strains to H_2_O_2_ and *o*-vanillin, cells were grown in 3 mL of YPD medium overnight, washed twice with phosphate-buffered saline (PBS; pH 7.4), and resuspended in PBS. Cell suspensions were spotted on YPD solid media with or without H_2_O_2_ and with or without *o*-vanillin. The plates were incubated at 30°C for 2 days and photographed.

### RNA Sequencing and Transcriptome Data Analysis

The wild-type strain was grown at 30°C overnight. The cells were transferred to 50 mL of YPD medium (final density of 1.0×10^6^ cells/mL) with 125 µg/mL *o*-vanillin (dissolved in dimethyl sulfoxide (DMSO)) or with DMSO (no drug control), incubated at 30°C for 3 h with shaking, and harvested for RNA extraction. Total RNA was extracted using the RiboPureTM-Yeast Kit (Ambion) following the manufacturer’s instructions. Contaminating genomic DNA was eliminated by RNase-free DNase (Invitrogen), the quantity and quality of total RNA were evaluated using RNA electropherograms (Agilent 2100 Bioanalyzer), and the RNA integrity numbers (RIN) were determined [18]. Total RNA (10 µg) from each sample was used as starting material to prepare sequencing libraries. mRNA was purified with the Dynabeads mRNA Purification Kit (Life Technologies), and the resulting mRNA was processed with the Illumina TruSeq RNA Sample Prep kit v2 (Illumina) following the manufacturer’s instructions. One lane per sample was used for sequencing with the Illumina Genome Analyzer IIx (Illumina) to generate nondirectional, single-ended 36-base pair reads. Quality-filtered reads were mapped to the reference genome sequence (http://www.broadinstitute.org/annotation/genome/cryptococcus_neoformans/MultiHome.html) using CLC Genomics Workbench 4.5 (CLC bio). The relative transcript abundance was computed by counting the reads per kilobase of exon model per million mapped sequence reads (RPKM) [19]. Processed data were deposited in the Gene Expression Omnibus (GEO) database with accession number GSE52645. Gene ontology (GO) analysis was performed by assigning GO categories to each gene according to the Gene Ontology Consortium database [20]. Each gene was compared to the GO database using BLASTP searches, and the best hit with an e-value lower than 10^−5^ and a hit coverage of more than 50% was adopted. A hit map based on log-transformed fold change values was generated by using TM4: MeV [21].

### Quantitative Real-time PCR

Primers for real-time PCR were designed using Primer Express software 3.0 (Applied Biosystems) and are listed in Table S2. Total RNA was extracted with the RiboPure-Yeast Kit (Ambion), and cDNA was synthesized using the RevertAid First Strand cDNA synthesis Kit (Fermentas). Relative gene expression was quantified using a 7500 system (Applied Biosystems) based on the 2^−△△Ct^ method. *TEF2* (CNAG_00044, translation elongation factor 2) was used as a reference.

### Fluorescence-activated Cell Sorting Analysis

To perform flow cytometric analysis, cells were grown in YPD at 30°C overnight. Cells were diluted to 1.0×10^7^ cells/mL in 4.5 mL of YPD containing 125 µg/mL *o*-vanillin and cultured at 30°C for an additional 2 h. After incubation, 1 mL of cells was washed twice with PBS (pH 7.4) and resuspended in 1 mL of YPD. Dihydrorhodamine 123, at a final concentration of 10 µg/mL, was added, and the cell suspension was incubated for an additional 30 min. A cell suspension of 50 µL was withdrawn and mixed with 2 mL of 50 mM Tris-HCl (pH 8.0). Flow cytometry was performed with 2×10^4^ cells, and the results were analyzed using the FL1 channel with FACSCalibur (BD Biosciences). The software Flowjo 8.7 was used to process images.

## Results

### The Vanillin Derivatives showed Antifungal Activity

**Figure 1:**
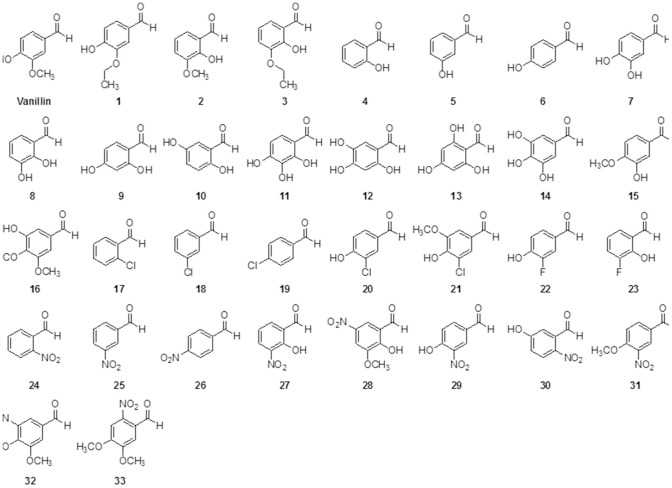
Vanillin and vanillin derivatives tested in this study. Compounds **1** to **16** are hydroxy and alkoxy benzaldehydes. Compounds **17** to **23** are halogenated benzaldehydes, and compounds **24** to **33** are nitrated benzaldehydes.

Thirty-three vanillin derivatives were classified into three groups: group A includes hydroxy and alkoxy benzaldehydes (**1** to **16**), group B includes halogenated benzaldehydes (**17** to **23**), and group C includes nitrated benzaldehydes (**24** to **33**) (Figure 1). To evaluate antifungal activity, we determined the MIC of each vanillin derivative. As previously reported, vanillin showed a relatively high MIC value against *C. neoformans*, suggesting its minimal antifungal activity [22]. In contrast, the derivatives in group A displayed relatively low MICs compared to that of vanillin. Among them, *o*-vanillin (**2**) and *o*-ethyl vanillin (**3**), specifically, showed the lowest MICs (4 µg/mL), which was even lower than that of fluconazole observed in our study, suggesting that *o*-vanillin and *o*-ethyl vanillin have strong antifungal activity against *C. neoformans* (Table 1). All other derivatives in group A displayed some degree of antifungal activity except for the monohydroxy benzaldehydes (**4,** **5, 13**, **15,** and **16**), indicating that hydroxy and alkoxy benzaldehydes are potent antifungal agents for treatment of cryptococcosis. In contrast, the vanillin derivatives in group B and group C mainly displayed significantly high MICs against *C. neoformans*, suggesting that halogenated and nitrated benzaldehydes have poor antifungal efficacy. These results indicated structural correlations between the vanillin derivatives and the antifungal activity against *C. neoformans*; thus, the hydroxyl or alkoxy group would be more advantageous than the halogenated or nitrated group in benzaldehyde as an antifungal agent.

**Table 1:** Antifungal activity of vanillin and 33 vanillin derivatives against *C. neoformans*.

Compound		MIC (µg/mL)
**Vanillin**		>128
Group A^1^	**1**	16
	**2**	4
	**3**	4
	**4**	>128
	**5**	>128
	**6**	32
	**7**	64
	**8**	8
	**9**	16
	**10**	8
	**11**	32
	**12**	16
	**13**	>128
	**14**	8
	**15**	>128
	**16**	>128
Group B^3^	**17**	>128
	**18**	>128
	**19**	>128
	**20**	>128
	**21**	>128
	**22**	>128
	**23**	16
Group C^2^	**24**	64
	**25**	128
	**26**	16
	**27**	128
	**28**	>128
	**29**	>128
	**30**	>128
	**31**	>128
	**32**	>128
	**33**	128
**Fluconazole**		8

1 Group A was for hydroxy and alkoxy benzaldehydes;

2 Group B was for halogenated benzaldehydes;

3 Group C was for nitrated benzaldehydes.

### *o*-Vanillin Significantly Altered the Global Transcript Profiles of *C. neoformans*

**Figure 2:**
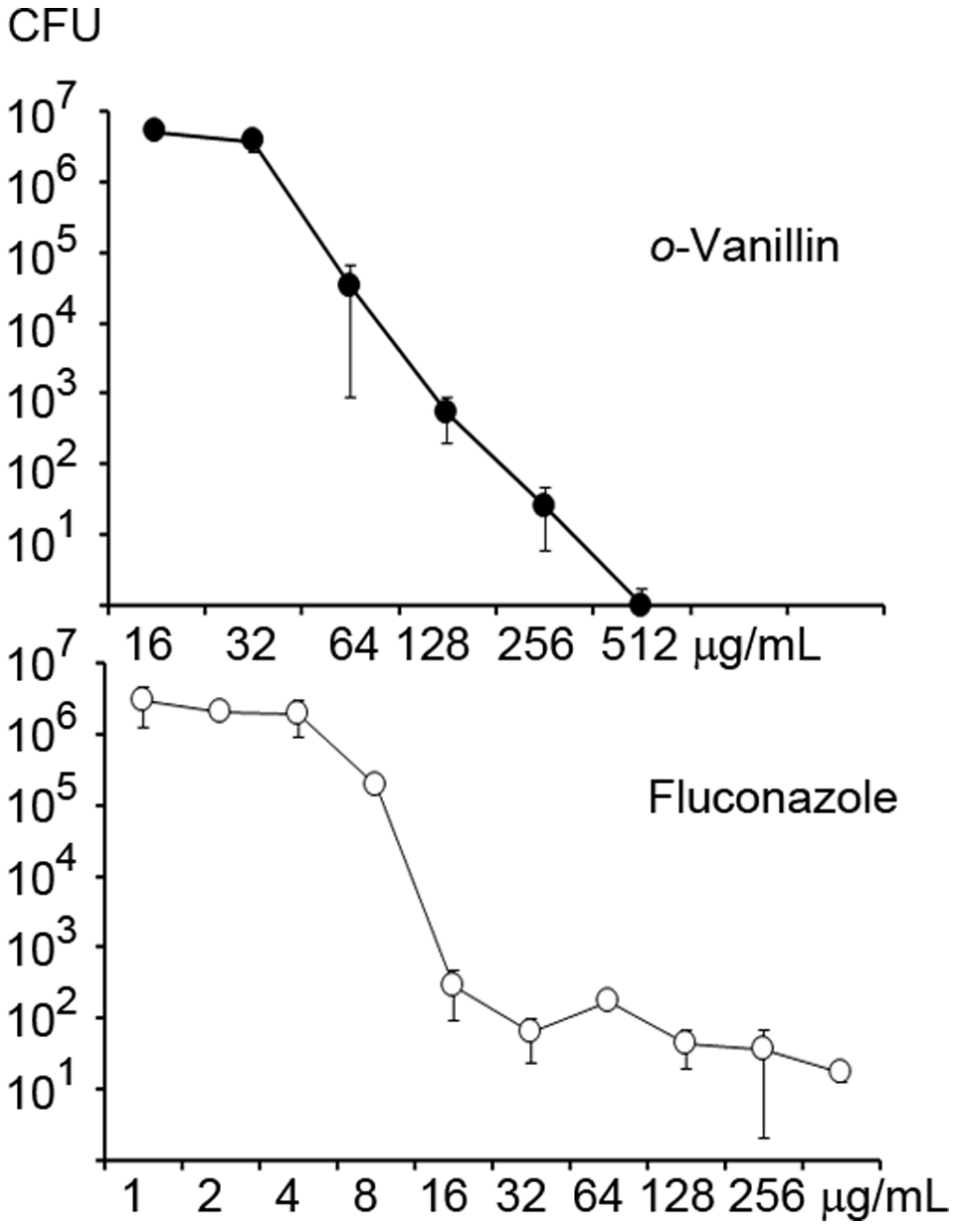
Viability of *C. neoformans* in the presence of *o*-vanillin. The fungal cells were grown in YPD media containing different concentrations of *o*-vanillin or fluconazole as indicated, and incubated at 30°C. Viability of the cells were determined by measuring CFUs after 2 days. Fluconazole was included as a control.

The vanillin derivative *o*-vanillin showed strong antifungal activity, and we selected this derivative to further study and determine the inhibitory mechanism against *C. neoformans*. We analyzed transcriptome changes in the fungal cells treated with *o*-vanillin using RNA-Seq. As mentioned above, transcriptome analysis has been successfully used in a number of other studies to define the target pathway and mechanism of action of antifungal drugs [11], [12], [23]. To determine the concentration of *o*-vanillin for the culture conditions for the transcriptome analysis, we analyzed the viability of the cells grown in the presence of the compound by measuring colony-forming units (CFUs). Interestingly, unlike fungistatic agent fluconazole, *o*-vanillin displayed fungicidal activity and no colony was observed at concentrations above 512 µg/mL (Figure 2). We should note that CFU and transcriptome analyses were performed using YPD medium rather than minimal RPMI medium, which was used for MIC determination in the present study. The final concentration of 128 µg/mL, at which viability was reduced by at least 50%, was selected as the concentration for the cultures for transcriptome analysis.

**Figure 3:**
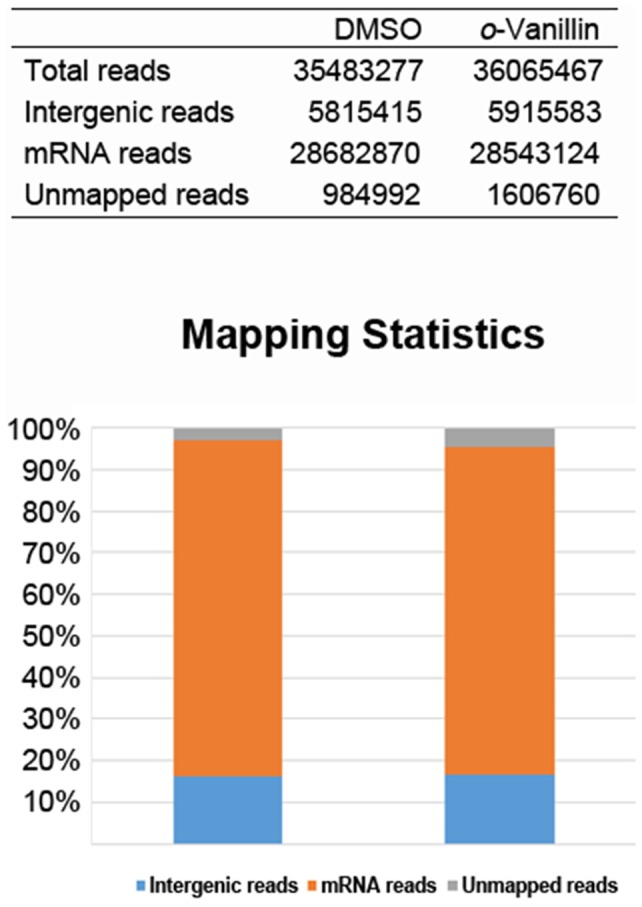
Sequence read mapping statistics. The number of the sequence reads mapped to the reference genome (*Cryptococcus neoformans* var. *grubii* H99). In total, 96.37% sequence reads were mapped against the genome of *C. neoformans*.

**Figure 4:**
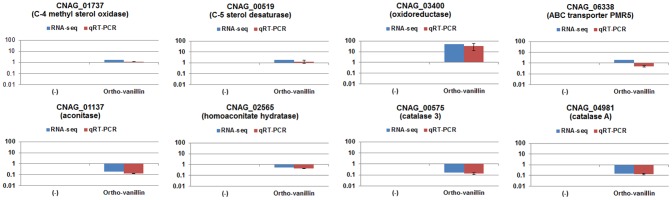
Confirmation of RNA-Seq data by quantitative RT-PCR (Q-RT-PCR). Eight genes were selected, and their transcript levels were quantified by Q-RT-PCR. Results of Q-RT-PCR are illustrated in parallel with the RNA-Seq data, and showed identical patterns. Data are averages from three independent experiments with three biological replicates, normalized by *TEF2* as an internal control, and presented as relative expression levels in comparison to the transcript levels in the cells cultured in the absence of *o*-vanillin. Bars represent standard deviations.

The wild-type *C. neoformans* cells were grown in the presence or absence of *o*-vanillin, and cDNA libraries were constructed using mRNA purified from each culture and sequenced (see Materials and Methods). In total, 71,548,744 sequence reads were obtained from the cDNA libraries, and of these, 68,956,992 (96.37%) sequence reads were mapped against the genome of *C. neoformans* var. *grubii* H99 (Figure 3). *o*-Vanillin treatment altered the transcript levels of 1,994 genes by more than 2-fold. Among the differentially expressed genes, 665 and 245 genes were up- and down-regulated by more than 3-fold, respectively, indicating the substantial effect of *o*-vanillin on the transcriptome of *C. neoformans* (Table S3). A subset of these differentially expressed genes were selected, and changes of their transcript levels were confirmed by quantitative real-time PCR (Q-RT-PCR) (Figure 4).

Our RNA-Seq analysis showed the transcript levels of 6,980 genes in the genome of *C. neoformans*. Among these, 4,067 genes were categorized based on GO terms. We used these GO terms, in particular biological processes, to define the possible target cellular processes of *o*-vanillin. We focused on GO terms that were highly enriched within the up-regulated genes upon treatment with *o*-vanillin because a number of previous transcriptome analyses suggested that the genes in the putative target pathway of an antifungal drug tend to be up-regulated upon treatment for compensation; for example, fluconazole treatment increases the transcript levels of the genes in the ergosterol biosynthesis pathway. As shown in Table 2, the GO term ‘oxidation-reduction process’ was the most highly enriched among the genes up-regulated by *o*-vanillin treatment, implying that the redox status in *C. neoformans* was substantially influenced by *o*-vanillin. We also observed that the genes with the GO terms ‘transmembrane transport’ and ‘proteolysis’ were highly enriched. The genes with transmembrane transport are mainly involved in efflux functions, suggesting that the fungal cells highly express membrane transporters to remove the toxic antifungal compound from the intracellular environment. In addition, enrichment of the GO term ‘proteolysis’ may indicate that the cells are under oxidative stress, since protein quality control and proteolysis mechanisms are involved in oxidative stress response in *Saccharomyces cerevisiae* [24].

**Table 2:** Enrichment of GO terms for the biological processes*.

GO Biological process	Count
oxidation-reduction process	38
transmembrane transport	38
proteolysis	14
carbohydrate metabolic process	13
regulation of transcription from RNA polymerase II promoter	12
regulation of transcription, DNA-dependent	9
pentose-phosphate shunt	5
acyl-carrier-protein biosynthetic process	3
biosynthetic process	3
transcription, DNA-dependent	3
transport	3

*Genes that were up-regulated more than 3-fold by *o*-vanillin treatment were analyzed for enrichment of GO terms of the biological processes. GO terms that contains at least 3 count (genes) were shown.

### *o*-Vanillin Triggered Oxidative Stress in *C. neoformans*

The results of the transcriptome analysis showed that the most significantly affected cellular process by *o*-vanillin is cellular redox homeostasis. An imbalance in cellular redox homeostasis is often caused by oxidative stress, and therefore, we hypothesized that a similar stress was triggered by *o*-vanillin in *C. neoformans*. We predicted that the *C. neoformans* mutants that showed increased sensitivity to oxidative stress would grow more slowly than the wild type in the presence of *o*-vanillin if the compound indeed induces a similar stress. To confirm this hypothesis, we constructed mutants lacking *SOD1* or *SOD2*, which encode copper-zinc-dependent cytosolic superoxide dismutase (CuZn-SOD) and manganese-dependent mitochondrial superoxide dismutase (Mn-SOD), respectively. The mutant lacking the gene *CFO1*, which encodes membrane ferrioxidase and is hypersensitive to oxidative stress due to reduced mitochondrial function, was also included in the experiment [23], [25]. Moreover, the mutant lacking *SIT1*, which encodes the siderophore transporter and was shown to be independent of oxidative stress defense, was included as a negative control strain [26].

**Figure 5:**
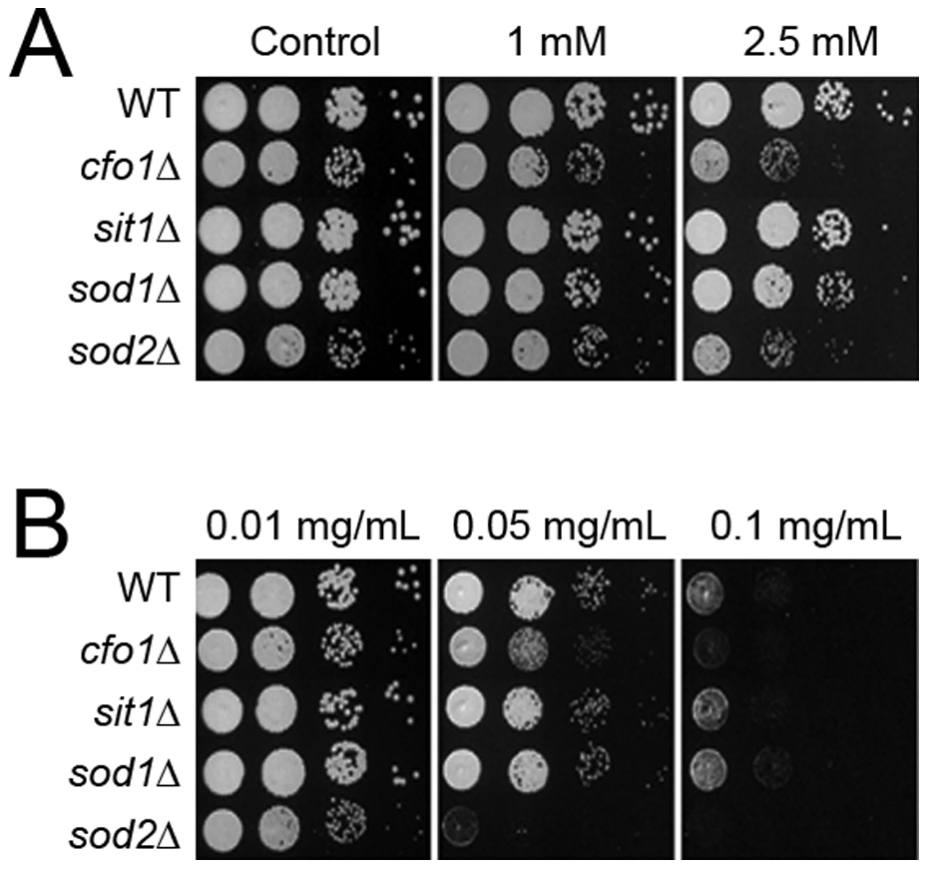
Mutants that have increased sensitivity to oxidative stress and reduced mitochondrial functions are highly sensitive to *o*-vanillin. The sensitivity of the wild type, *cfo1* mutant, and *sod2* mutant to H_2_O_2_ (A) and *o*-vanillin (B) was investigated by monitoring the growth of the strains in media containing each compound. Cells at 10-fold serial dilutions (starting at 10^4^ cells) were spotted onto plates, incubated at 30°C for 2 days, and photographed.

The growth of the mutants lacking *SOD1*, *SOD2*, *CFO1*, or *SIT1* in the presence of hydrogen peroxide (H_2_O_2_; an oxidative stress-inducing agent) or *o*-vanillin was monitored. We first confirmed the results of a previous report that showed that hydrogen peroxide significantly inhibits the growth of both the *sod2* mutant and the *cfo1* mutant (Figure 5A) [23], [27]. The growth of the *sod1* mutant and the *sit1* mutant was not inhibited, as previously reported [26]. We next challenged the same mutant sets with *o*-vanillin and found that the *sod2* mutant and the *cfo1* mutant displayed significantly increased sensitivity to the compound, while the *sod1* mutant and the *sit1* mutant showed no changes in growth phenotype compared to the wild type (Figure 5B). These results suggest that *o*-vanillin triggers oxidative stress, which leads to the disruption of cellular redox homeostasis in *C. neoformans*.

### *o*-Vanillin Caused Mitochondrial Dysfunction in *C. neoformans*

Previously, several independent studies suggested that the *sod2* mutant and the *cfo1* mutant had substantial depletion of mitochondrial functions, which mainly accounts for the increased susceptibility of the mutants to oxidative stress [23], [25]. We therefore analyzed the obtained transcriptome data focusing on the genes that were predicted to encode mitochondrial proteins. The *C. neoformans* genome contains at least 221 genes that are predicted to encode mitochondrial proteins, and the transcriptome data showed that many of these genes were differentially expressed following *o*-vanillin treatment. Among these 221 genes, the transcript levels of 62 genes were altered by *o*-vanillin treatment by at least 2-fold (Table S4), which further confirmed our hypothesis that *o*-vanillin substantially influences mitochondrial functions in *C. neoformans*.

**Figure 6:**
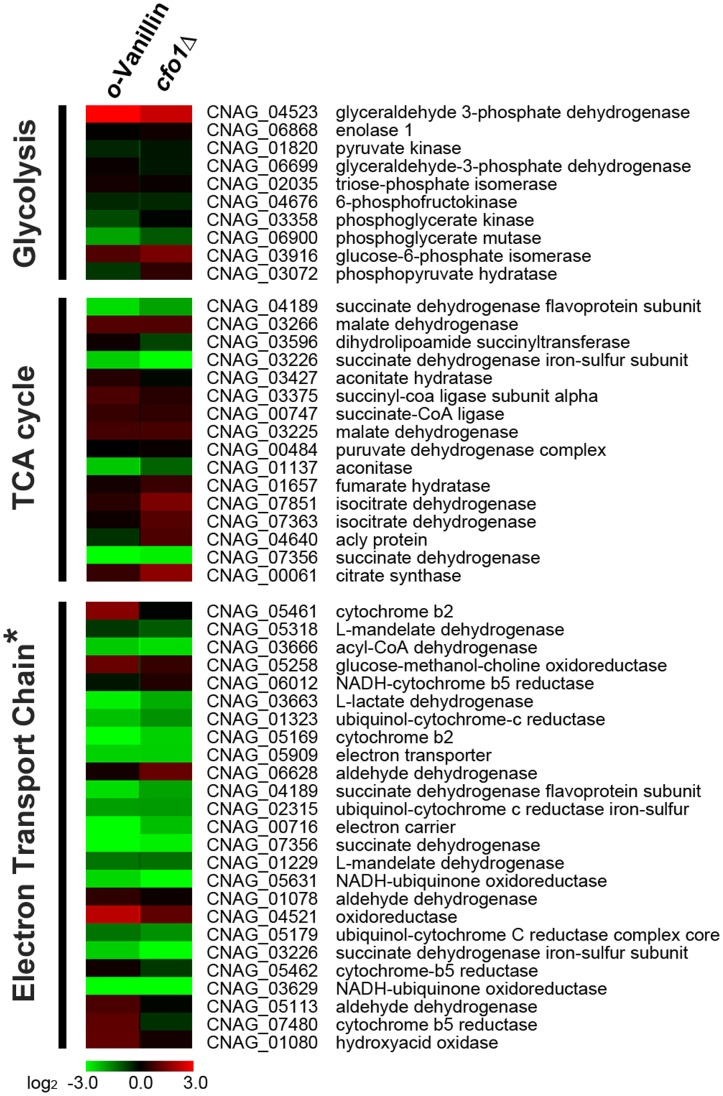
*o*-Vanillin causes differential expression of the genes involved in respiration. Genes related to cellular respiration (glycolysis, the TCA cycle, and the electron transport chain) were selected from our transcriptome data and compared with the previous transcriptome data derived from a study with the *cfo1* mutant. The results are represented by the hip-map, and the asterisk indicates that the genes required for the electron transport chain were more significantly down-regulated by *o*-vanillin.

One of the main cellular functions of mitochondria is energy production by oxidative respiration through the tricarboxylic acid (TCA) cycle and the electron transport chain. We predicted that the TCA cycle and the electron transport chain might be significantly distorted if the mitochondrial functions were affected by *o*-vanillin treatment. To confirm this hypothesis, we specifically focused on alterations in transcript levels of the genes encoding proteins required for the TCA cycle and the electron transport chain. As expected, the transcript levels of the genes required for both the TCA cycle and the electron transport chain were significantly reduced by *o*-vanillin. In particular, the genes required for the electron transport chain showed profound down-regulation, suggesting that *o*-vanillin mainly affects the mitochondrial electron transport chain. We also took advantage of the previously reported transcriptome data of the *cfo1* mutant, which showed significantly reduced mitochondrial respiration [23]. The obtained transcript profiles of the genes required for respiration were aligned with the previously derived transcriptome data from the *cfo1* mutant, and a clustering analysis was performed to compare the patterns of changes in the transcript levels of the genes. Results of this analysis showed highly similar patterns of down-regulation in the genes involved in the TCA cycle and the electron transport chain between the *o*-vanillin treated cells and the *cfo1* mutant (Figure 6).

**Figure 7:**
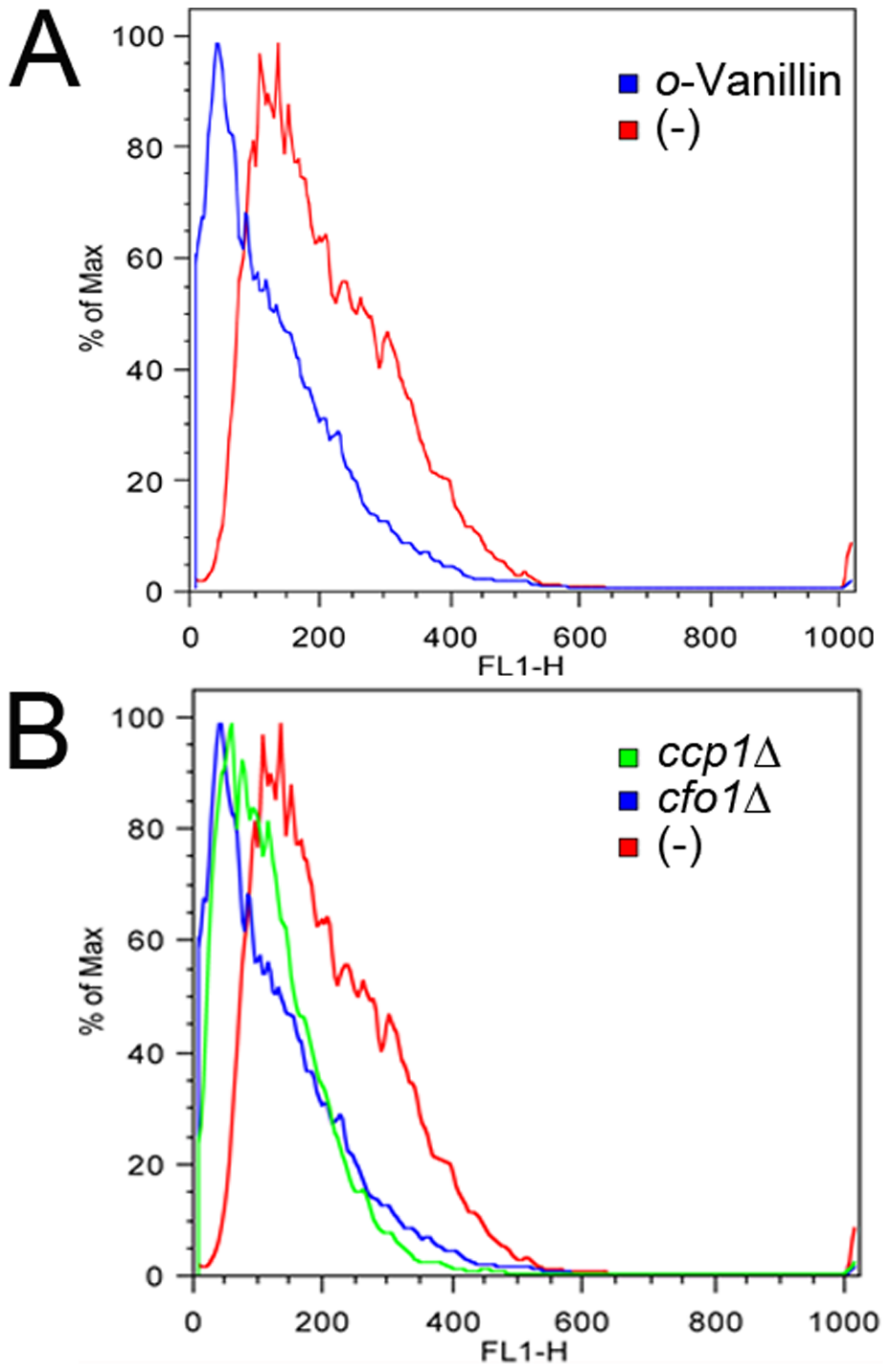
Mitochondrial functions were altered by *o*-vanillin treatment. (A) The results of FACS analysis showed that dihydrorhodamine 123 accumulated less in the *o*-vanillin treated cells than in the untreated cells. (B) The *cfo1* mutant and the *ccp1* mutant, which were shown to have abnormal mitochondrial functions in previous studies [23], [28], also showed less accumulation of dihydrorhodamine 123 and were included as controls.

We further investigated depletion of mitochondrial function by *o*-vanillin using dihydrorhodamine 123, which is a fluorescent dye that stains mitochondria in living cells. The damaged mitochondria retained much less dihydrorhodamine 123 than did the healthy mitochondria. We treated the fungal cells with *o*-vanillin and dihydrorhodamine 123 and analyzed the fluorescence intensity by fluorescence-activated cell sorting (FACS). The observation of fluorescence profiles indicated that dihydrorhodamine 123 accumulated less in the cells treated with *o*-vanillin than in untreated cells, suggesting that mitochondrial functions were damaged by the compound (Figure 7A). Our results were also supported by the observation of less accumulation of dihydrorhodamine 123 in the *cfo1* mutant and the *ccp1* mutant (Figure 7B), which displayed significantly reduced mitochondrial function. Taken together, the results of the present study suggest that *o*-vanillin has antifungal activity against *C. neoformans* and substantially distorts mitochondrial functions, resulting in an overall deficiency of oxidative stress defense mechanisms in *C. neoformans*. Therefore, *o*-vanillin can be an effective drug candidate to treat cryptococcosis.

## Discussion

In this study, we investigated the antifungal activity of vanillin and its derivatives, which were classified into three groups (hydroxy and alkoxy benzaldehydes, halogenated benzaldehydes, and nitrated benzaldehydes), and determined their antifungal activity against *C. neoformans*. Our results suggested a structural correlation between the compounds and antifungal activity. Most of the hydroxy and alkoxy benzaldehydes showed strong antifungal activity, while the halogenated benzaldehyde and nitrated benzaldehyde compounds showed no activity. *o*-Vanillin and *o*-ethyl vanillin belong to the hydroxy and alkoxy benzaldehydes group, and showed the highest antifungal activity among all tested compounds of this group. We used *o*-vanillin to further study the mechanism of antifungal action. We obtained global transcript profiles of the wild type cells grown in the presence or absence of *o*-vanillin by RNA Seq, and analyzed the data using GO functional categories. The results of the transcriptome analysis revealed that *o*-vanillin mainly disrupts redox homeostasis in *C. neoformans*, and that the fungal cells undergo oxidative stress induced by the compound.

Reactive oxygen species (ROS), such as superoxide anion (O^⋅^_2_^−^), hydroxyl radical (OH^⋅^), and hydrogen peroxide (H_2_O_2_), are byproducts of aerobic respiration; they trigger oxidative stress, which detrimentally affects multiple cellular processes. ROS are also involved in host-pathogenic microbe interactions. The host phagocyte produces ROS to inhibit and remove the intracellular pathogens; however, pathogenic microbes have well-developed defense mechanisms to survive under oxidative stress. *C. neoformans* is no exception, and the fungus utilizes enzymes such as superoxide dismutases, cytochrome c peroxidase, and thiol-peroxidases to cope with oxidative stress within the host phagocyte [27]–[30]. Oxidative stress within mitochondria has also been reported to increase the sensitivity of the fungus to azole antifungal drugs [23]. Thus, the oxidative stress response and mitochondrial functions play critical roles in the physiology and pathogenesis of *C. neoformans*. The present study revealed that the fungicidal activity of *o*-vanillin involves the induction of oxidative stress and reduced mitochondrial functions, which is supported by our transcriptome analysis results and phenotypic observations of the mutants lacking *SOD2* and *CFO1*. In *C. neoformans*, mitochondrial Mn-SOD, which is encoded by *SOD2*, has been suggested to be responsible for redox homeostasis in mitochondria, and the absence of Mn-SOD activity causes substantial damage to mitochondrial DNA, proteins, and lipids. Indeed, the *sod2* mutant showed increased sensitivity to oxidative stress, reduced growth at the host body temperature of 37°C compared to the wild type, and avirulence in a murine inhalation model of cryptococcosis [27]. Mitochondrial functions were also influenced by deletion of *CFO1* in *C. neoformans*. The mutant lacking *CFO1* showed reduced mitochondrial respiration and increased sensitivity to oxidative stress and azole antifungal drugs [23]. These previous findings and the results from this study strongly support our conclusion that *o*-vanillin induces oxidative stress and disrupts mitochondria functions in *C. neoformans*. Interestingly, a previous study by Kim et al. [31] showed that vanillin and the vanillin derivatives vanillic acid and vanillyl acetone also trigger oxidative stress within mitochondria and inhibit the growth of *S. cerevisiae*, *Aspegillus flavus*, and *A. fumigatus*.

Despite its complex nature, the fungal mitochondrion has been considered an effective drug target for treatment of fungal infections. *C. neoformans* and *C. albicans*, the most prevalent human fungal pathogens, are petite negative, and a number of studies suggested tight connections between mitochondrial functions and virulence in these fungi. As mentioned above, the mutant lacking *SOD2*, which encodes mitochondrial Mn-SOD, showed reduced virulence [25]. Moreover, global transcriptome analysis by serial analysis of gene expression (SAGE) during colonization of *C. neoformans* in the host central nervous system showed that an increase in mitochondrial respiration functions is required for disease progression [32]. The involvement of mitochondria in virulence was also reported in a study with another *Cryptococcus* species, *C. gattii*, which caused the Vancouver Island and North American outbreaks, and typically infects immunocompetent individuals. Hypervirulent *C. gattii* strains were found to have high expression of genes within the mitochondrial genome and up-regulated mitochondrial functions [33]. Similarly, several studies showed the effects of dysfunctional mitochondria in *C. albicans*. For example, deficiency of mitochondrial functions by deletion of the mitochondrial protein-encoding gene *GOA1* in *C. albicans* caused not only decreased respiration and mitochondrial membrane potential but also loss of virulence in a murine model of disseminated candidiasis [34]. Furthermore, the absence of Sam37, which is the mitochondrial outer membrane sorting and assembly machinery (SAM) complex subunit, rendered *C. albicans* avirulent [35]. In addition, the close association between cell wall and membrane integrity makes mitochondrial functions an attractive target for novel antifungal treatment. Tetracyclin treatment caused dysfunction of mitochondria, which reduced ergosterol levels in the cell membrane and thus increased the sensitivity of *C. neoformans* and *C. albicans* to amphotericin B [36]. The influence of mitochondrial deficiency on cell wall integrity was also suggested by a study evaluating a collection of *S. cerevisiae* mitochondrial mutants and a *C. albicans* mutant lacking *CCR4*/*POP2*, which encodes mRNA deadenylase and regulates mitochondrial functions and phospholipid homeostasis [37]. Several drug candidates that inhibit mitochondrial functions in fungi have been proposed. An amino acid-derived 1,2-benzisothiazolinone (DFD-VI-15) showed inhibitory effects on fungal mitochondria, and the compound showed fungicidal activity against *C. neoformans* and *C. albicans* [38]. Other compounds that inhibit mitochondrial functions of the pathogenic fungi include 3-hydroxy oxylipins, acetylsalicylic acid, and the arylamidine compound T-2307 [39], [40]. These previous studies investigating a series of antifungal drug candidates demonstrated that mitochondria are an attractive antifungal target, and support our conclusion that *o*-vanillin can be an effective antifungal drug candidate. *o*-Vanillin triggered mutations in *Escherichia coli* when it was treated with *N*-methyl-*N*’-nitro-*N*-nitrosoguanidine (MNNG) or *N*-methyl-*N*-nitrosourea [41], and treatment of Chinese hamster ovary cells with 400 µg/mL *o*-vanillin for 2 h in the presence of MNNG caused structural chromosome aberrations [42]. However, possible toxic effects could be eliminated because *o*-vanillin itself is not a clastogen, and thus no toxicity of the compound itself has been reported [42].

## Supporting Information

Table S1 Primers used to construct the *sod1* mutant, the *sod2* mutant and the *ccp1* mutant.
(DOCX)

Table S2 Primers used for Q-RT-PCR.
(DOCX)

Table S3 Results of RNA Seq analysis.
(XLSX)

Table S4 Differential expression of the genes encoding mitochondrial proteins.
(XLSX)
