# Supplementary material for: A Vanillin Derivative Causes Mitochondrial Dysfunction and Triggers Oxidative Stress in Cryptococcus neoformans
Source: PLoS One. 2014 Feb 20;9(2):e89122. doi: 10.1371/journal.pone.0089122 (PMC3930674; doi:10.1371/journal.pone.0089122)
Supplement: Table S1 — Primers used to construct the sod1 mutant, the sod2 mutant and the ccp1 mutant. (DOCX) [file pone.0089122.s001.docx]

**TableS1. Primers used to construct the *sod1* mutant, the *sod2* mutant and the *ccp1* mutant.**

| **Primers** | **Sequence** |
| --- | --- |
| SOD1_KO1 | CGCTGGAGGAAGAGAAAGATGATAACGC |
| SOD1_KO2 | AATTCTGCAGATATCCATCACACTGGCGGCTCGGTGGAGTTGTGCCGAGTAAGAAGAC |
| SOD1_KO3 | AATTCCAGCACACTGGCGGCCGTTACTAGTCCATCATTGGTCGAAGCCTCGTCGTCCA |
| SOD1_KO4 | CTTGAACCTCCTGAACGGGCTCTG |
| SOD1_KO5 | AAACAGGACGAGGCAGAAGATCAG |
| SOD1_KO6 | CAATAGGGTCGTTGATAGCGGACATGG |
| SOD2_KO1 | GAGGGATACTCACCCAAGTAGAGAACAGC |
| SOD2_KO2 | AATTCTGCAGATATCCATCACACTGGCGGCGGGAAATAGCAGTCTTGAAGTCGCCATCAG |
| SOD2_KO3 | AATTCCAGCACACTGGCGGCCGTTACTAGTATTGTCTATCTTCGGGTGTGGCACAG |
| SOD2_KO4 | GAGAGTGGAGACGAGAGAAAGCATTG |
| SOD2_KO5 | CTCCATAGAGTGTCTCGTCCATAGCCATAC |
| SOD2_KO6 | TTACCAGGGCTAACGCCTCGTTACTG |
| CCP1_KO1 | TAGGGAGAAGGTGCTAGACTTCCGTTAC |
| CCP1_KO2 | AATTCTGCAGATATCCATCACACTGGCGGCGTCGGCGACATATCAACTTAGCCTTGTG |
| CCP1_KO3 | AATTCTGCAGATATCCATCACACTGGCGGCGTTAATGTCCTTGCTGTTCGTTGTTTGACC |
| CCP1_KO4 | TGGTACACGTCATATCGCCGACAAAGAC |
| CCP1_KO5 | GATGTTGTTGCATCATGCTTAATGGCAC |
| CCP1_KO6 | CCGCACCATGATACTATGACGTGTATTTAC |
