# Supplementary material for: A Vanillin Derivative Causes Mitochondrial Dysfunction and Triggers Oxidative Stress in Cryptococcus neoformans
Source: PLoS One. 2014 Feb 20;9(2):e89122. doi: 10.1371/journal.pone.0089122 (PMC3930674; doi:10.1371/journal.pone.0089122)
Supplement: Table S2 — Primers used for Q-RT-PCR. (DOCX) [file pone.0089122.s002.docx]

**TableS2. Primers used for Q-RT-PCR.**

| **Primers** | **Sequence** |
| --- | --- |
| CNAG_00519_ qRT-PCR.F | CTTCCCCATCCCCCTGTT |
| CNAG_00519_ qRT-PCR.R | CCGGAAGAGCAGTGGTGTTT |
| CNAG_00575_ qRT-PCR.F | ATGTCATTCATGCCGTCCAA |
| CNAG_00575_ qRT-PCR.R | AGCTGTCTGGGCTTGAGGAA |
| CNAG_01137_ qRT-PCR.F | TCTCATGATGATTGGCACTGATT |
| CNAG_01137_qRT-PCR.R | CGCAAGCAACCATACCAAGA |
| CNAG_01737_qRT-PCR.F | CACCACCGCGCAAAACA |
| CNAG_01737_qRT-PCR.R | CCAGTTGAGTGCGGCAAAG |
| CNAG_02565_qRT-PCR.F | CGGTGGCGTCGGATGT |
| CNAG_02565_qRT-PCR.R | CCAAATACCAGCGGCATCA |
| CNAG_04981_qRT-PCR.F | AAGGTTGTCCCTGCATATGTCA |
| CNAG_04981_qRT-PCR.R | CAGCGCTGCCTCGTGAA |
| CNAG_06338_qRT-PCR.F | CAAGCCGATATCCACGAACCT |
| CNAG_06338_qRT-PCR.R | CGAAGCAAGGCGGAGAACT |
| CNAG_00044_qRT-PCR.F | CCTTCCTTGCCCTCTTCTCAT |
| CNAG_00044_qRT-PCR.R | AGCGACGACAGGGACAATG |
